# The monoclonal antibody EPR1614Y against the stem cell biomarker keratin K15 lacks specificity and reacts with other keratins

**DOI:** 10.1038/s41598-018-38163-5

**Published:** 2019-02-13

**Authors:** Hebah Aldehlawi, Katarzyna A. Niemiec, Deepa R. Avisetti, Anand Lalli, Muy-Teck Teh, Ahmad Waseem

**Affiliations:** Centre for Immunobiology and Regenerative Medicine, Institute of Dentistry, Barts and The London School of Medicine and Dentistry, Turner Street, London, E1 2AD United Kingdom

## Abstract

Keratin 15 (K15), a type I keratin, which pairs with K5 in epidermis, has been used extensively as a biomarker for stem cells. Two commercial antibodies, LHK15, a mouse monoclonal and EPR1614Y, a rabbit monoclonal, have been widely employed to study K15 expression. Here we report differential reactivity of these antibodies on epithelial cells and tissue sections. Although the two antibodies specifically recognised K15 on western blot, they reacted differently on skin sections and cell lines. LHK15 reacted in patches, whereas EPR1614Y reacted homogenously with the basal keratinocytes in skin sections. In cultured cells, LHK15 did not react with K15 deficient NEB-1, KEB-11, MCF-7 and SW13 cells expressing only exogenous K8 and K18 but reacted when these cells were transduced with K15. On the other hand, EPR1614Y reacted with these cells even though they were devoid of K15. Taken together these results suggest that EPR1614Y recognises a conformational epitope on keratin filaments which can be reconstituted by other keratins as well as by K15. In conclusion, this report highlights that all commercially available antibodies may not be equally specific in identifying the K15 positive stem cell.

## Introduction

The epidermis is a multilayered stratified epithelium designed to provide a protective barrier throughout the life of an individual. It is made up of two compartments, a basal cell compartment where cells are attached to the basal lamina and are mostly proliferating, and the suprabasal compartment where the progenies of the basal layer undergo differentiation. Epidermal basal keratinocytes predominantly express keratin 14 (K14), a type I keratin, which together with keratin 5 (K5), a type II keratin, assemble into intermediate filaments (IFs)^1,2^. In addition to K5/K14, the basal keratinocytes also express K15, which does not have a defined type II keratin partner and pairs with K5^3,4^. Synthesis of K5/K14 ceases when the committed cells in the basal layer move into the suprabasal layers but their expression continues in keratinocytes of the spinous layers^5–7^. The synthesis of K15 (mRNA and protein) on the other hand is confined only in the epidermal basal layer^8,9^. The downregulation of K5/K14/K15 synthesis in the spinous layer is accompanied by upregulation of differentiation-specific keratins K1 and K10. As the cells move further up into the stratum granulosum another type II keratin, K2, is induced^10,11^. This programme produces several layers of keratinocytes at different stages of differentiation until the cells are terminally differentiated and sloughed from the skin surface.

The balance between the proliferation and differentiation is important to establish the tissue homeostasis essential for the protective function of the epidermis. The epidermis is regenerated and maintained by stem cells present in the basal layer. Earlier reports had suggested that less than 10% of basal cells were stem cells in murine skin^12–15^, however, more recently this number has been revised to about 1 stem cell per 10,000 (0.01%) basal keratinocytes in interfollicular epidermis^16^. These stem cells can divide either symmetrically to produce two stem cells^17–19^, one of them later becomes a transit-amplifying (TA) cell, or divide asymmetrically (laterally or perpendicularly) to produce two different stem cells, one of them remains in the basal layer and the other is committed to undergo differentiation^20,21^. The TA cells in the symmetrical model divide rapidly only a few times to produce a population of “committed cells”, which become less adhesive due to down-regulation of integrin extracellular matrix receptors (reviewed in^18,22^) and leave the basal layer to move up into the spinous layer to begin the programme of differentiation. This can be followed precisely by expression of different keratins.

As stem cells in the basal layer play a key role in tissue regeneration and homeostasis, their precise identification and characterisation is important. Earlier studies exploited the slow cycling nature of these cells to develop label-retaining assays for their identification. In this assay all the S-phase cycling cells of the skin are first labelled with 5-bromo-2′-deoxyuridine (BrdU) or 3[H]-thymidine and the label is then chased for several weeks or months, the differentiating cells are lost from the skin surface, and the more proliferative cells dilute their label as they divide, leaving behind the slow cycling label-retaining cells (LRCs) as stem cells^13,14,23,24^. However, the cumbersome and time-consuming nature of these assays encouraged researchers to identify biomarkers which would specifically target stem cells. One of them, keratin K15, has received considerable attention as a biomarker of stem cells in stratified epithelia for the following reasons: first, localisation of K15+ cells in the murine and human hair follicle bulge region considered rich in multipotent stem cells^8,25^, second, K15 promoter was able to target β-galactosidase to the bulge region in murine epidermis^26^, third, K15 expressing murine bulge cells were able to reconstitute the entire epidermis and had higher proliferation potential than other keratinocytes^27^, fourth, K15+ epidermal cells were able to form fewer but larger colonies^28^, fifth, K15+ progenitor cells contribute towards homeostasis and regeneration in oesophagus^29^, and sixth, K15+ intestinal crypt cells were radio resistant and tumour initiating^30^. Any conclusion correlating the number or stemness of stem cell population in a tissue or in cultured cells with K15 expression is highly dependent on the specificity of the reagent used to detect the K15 polypeptide.

A number of mono- and polyclonal antibodies have been developed for detection of K15 in cultured cells and tissues using immunochemistry. The first monospecific K15 antibody was a polyclonal antibody reported by Lloyd and co-workers in 1995 against the last 12 residues of the K15 polypeptide^3^. Later, a mouse monoclonal antibody, LHK15, raised against the last 17 residues of K15 polypeptide had been published^8^ and is now commercially available. Two more mouse monoclonal against K15, clones 6B4F8 and 6E7, are commercially available but they have not been cited so far in any published study. In addition, two cross reacting mouse monoclonals, one against the C-terminal cytoplasmic domain of the cluster of differentiation 8 (CD8) polypeptide (C8/144B), and another against the N-terminal K18 peptide (LC18N) have been published^9,25^. More recently a rabbit monoclonal antibody, EPR1614Y, against a much longer epitope on K15, raised using a patented technology has been marketed by Abcam UK.

In this study we have systematically compared the specificities of two of the most widely used monoclonal antibodies, LHK15 and EPR1614Y, to detect K15 in cells and tissues. Although both antibodies recognise denatured K15, they react differently with the protein in tissues and cell lines. While LHK15 reacted specifically with keratin cytoskeleton containing K15, EPR1614Y recognised IFs completely devoid of K15. Our data suggests that EPR1614Y epitope may acquire a conformation that can also be mimicked by keratins other than K15.

## Results

**Figure 1:**
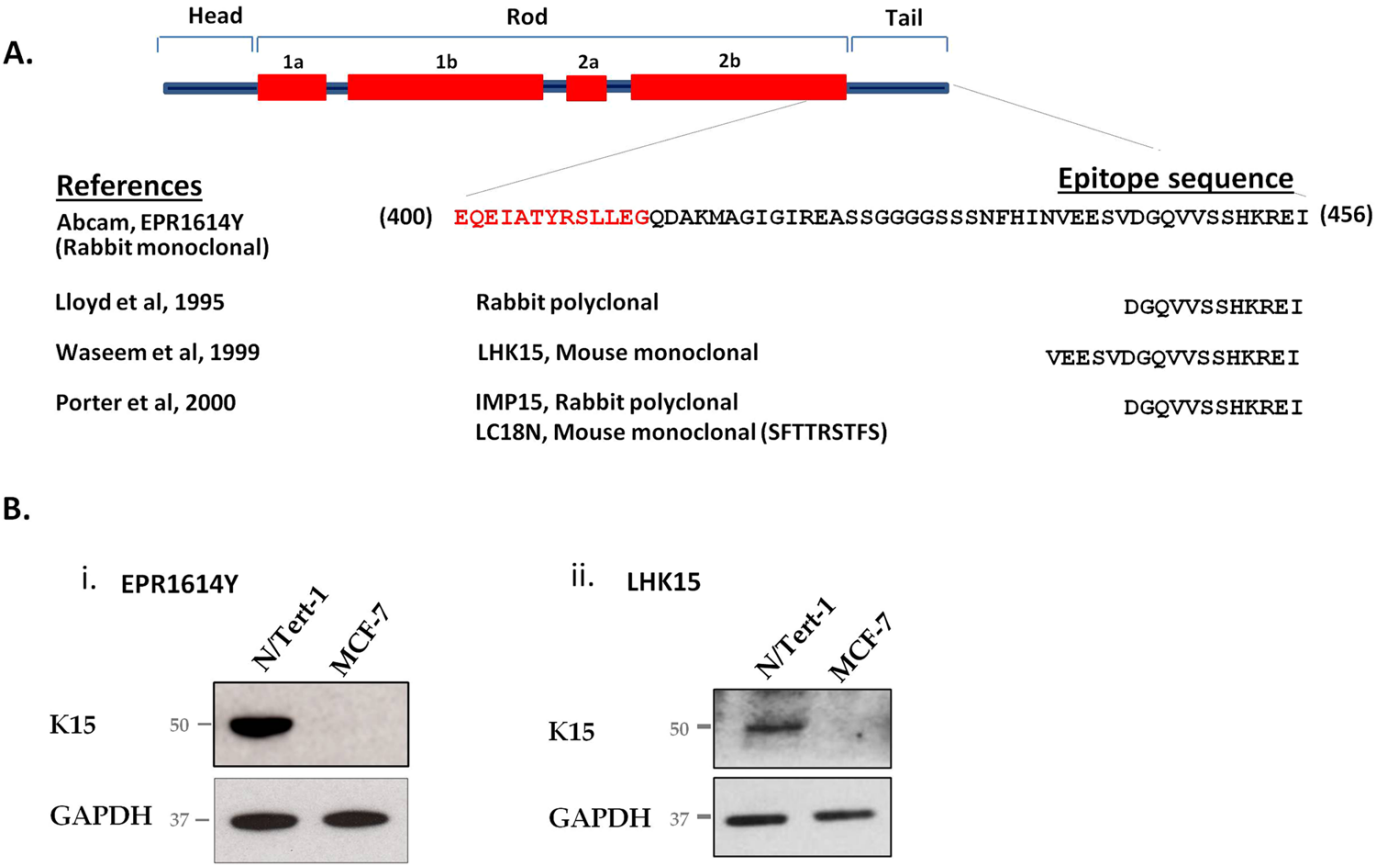
Location of antibody epitopes on K15 polypeptide. (**A**) The domain structure of an intermediate filament polypeptide is shown at the top. The sequence from 400–456 residues, which included the peptide used to raise EPR1614Y, including the end of rod (shown in red) and the entire tail domain of K15 is shown. The peptide sequences used to raise different antibodies along with published references are also listed. A keratin K18 peptide from the N-terminus used to raise LC18N antibody which cross reacts with K15 is also shown. (**B**) Western blotting of keratins present in N/Tert-1 and MCF-7 lysates using EPR1614Y (i) and LHK15 (ii). GAPDH was used as loading control. Relevant bands were cropped from different blots and grouped together. Original blots are shown in supplementary Fig. S1.

The epitope locations of 4 different K15 antibodies on the K15 polypeptide are shown in Fig. 1A. Two of them, LHK15 and EPR1614Y, are monoclonal and both are commercially available. The EPR1614Y epitope is located between 400–456 residues on K15 polypeptide (Abcam, UK; www.abcam.com/cytokeratin-15-antibody-epr1614y-ab52816.html) and therefore could overlap with the last 13 residues of the rod domain, on the other hand LHK15 has its epitope located in the last 17 residues of the tail domain^8^. We designed a series of experiments to compare the reactivities of EPR1614Y and LHK15 on cells and tissue sections. On western blotting using lysates derived from N/Tert-1, a K15+ cell line^31^ and MCF-7, a K15- human breast carcinoma cell line^32^, grown in 2-dimensional (2-D) cultures, both antibodies recognised a single 50 kDa band only in N/Tert-1 but not in MCF-7 cells (Fig. 1B). This set of experiments established that on western blot both antibodies were specifically reacting with the K15 polypeptide.

### Immunoreactivity of LHK15 and EPR1614Y with basal keratinocytes in skin

**Figure 2:**
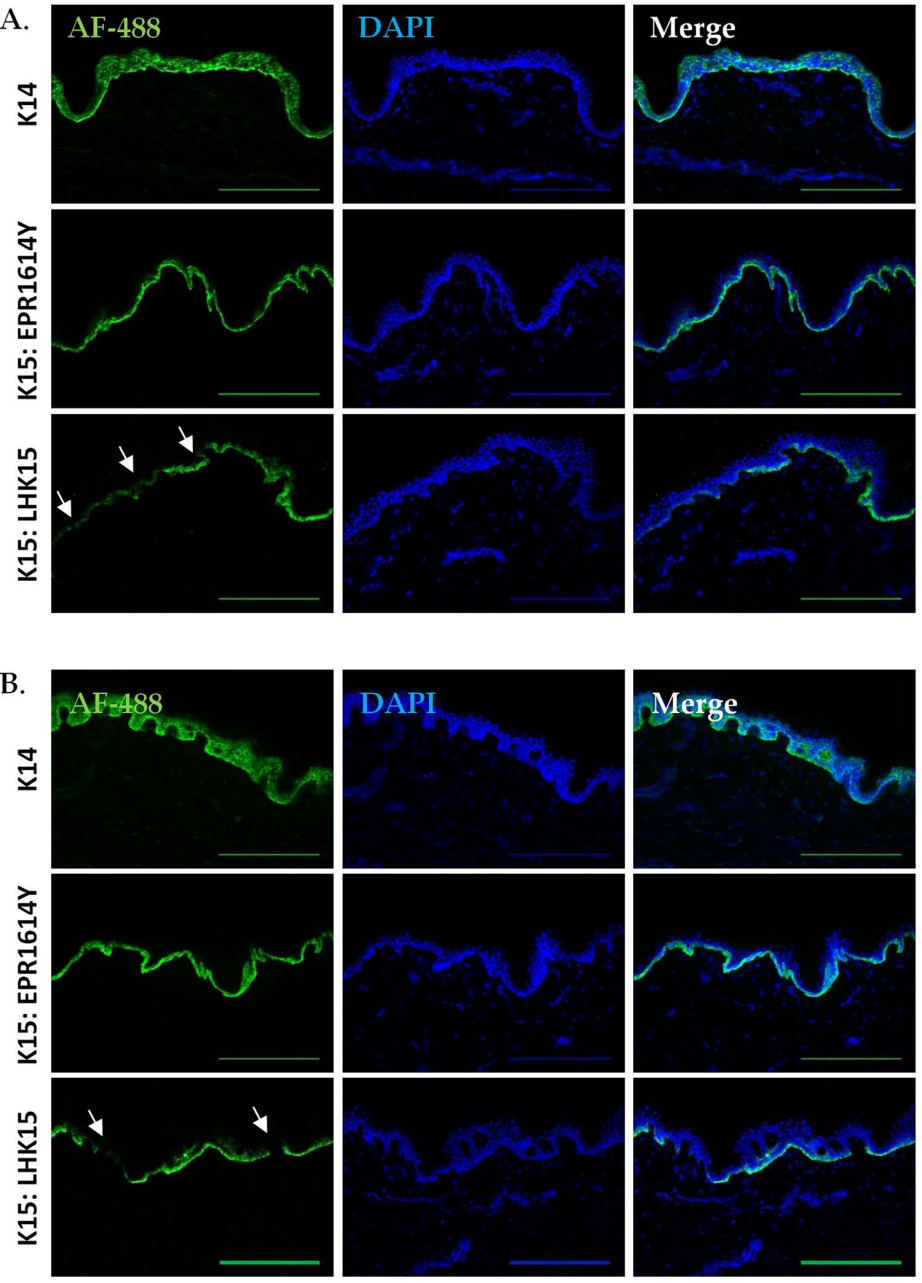
Specificity of anti-K15 antibodies on skin sections. Frozen human foreskin (**A**) and abdominal skin (**B**) sections were immuostained for K14 (LLOO1) and K15 (LHK15 and EPR1614Y) and the nuclei were counterstained with DAPI. Arrows indicate interruption in basal staining. Images were acquired using the Leica DM5000B epi-fluorescence microscope; scale bar = 250 μm.

To compare the reactivity of the two antibodies in skin tissues, sections of freshly frozen human foreskin and abdominal skin were immunostained with LHK15 and EPR1614Y. As shown in Fig. 2 both antibodies specifically reacted with the basal layer; EPR1614Y displayed homogenous reactivity with the basal keratinocytes whereas LHK15 showed interrupted staining in sections of both foreskin and abdominal skin. We also compared the K15 staining with K14, a known basal cell specific marker, using anti-K14 LLOO1 antibody, which reacted with basal and some suprabasal keratinocytes in both types of skin samples (Fig. 2A,B).

### Reactivity of LHK15 and EPR1614Y with keratin cytoskeleton in 2-D cultures

**Figure 3:**
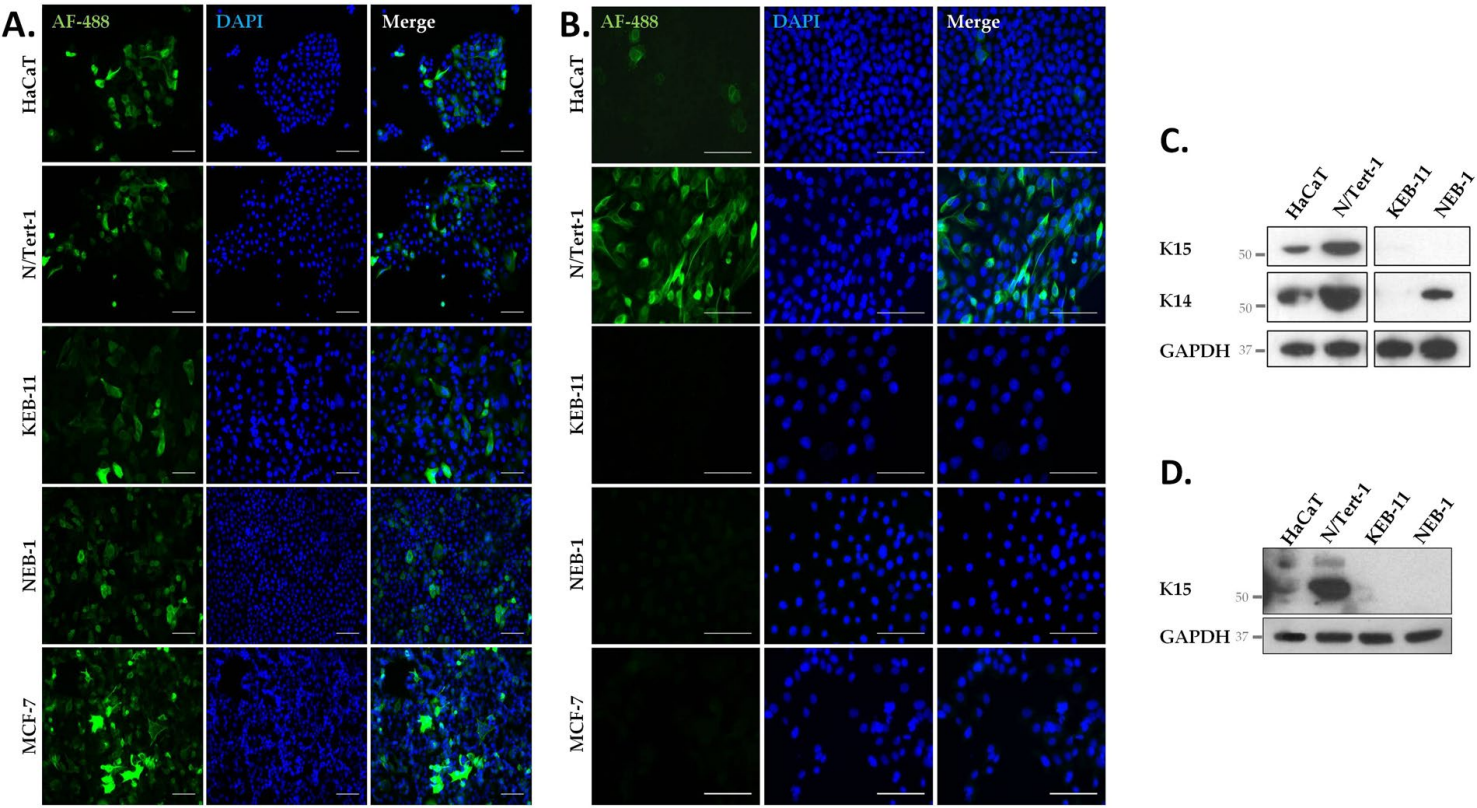
Immunoreactivity of EPR1614Y and LHK15 with keratins in epithelial cell lines. Cultured cells (HaCaT, N/Tert-1, KEB-11, NEB-1 and MCF-7) were fixed and stained for K15 (EPR1614Y) (**A**), K15 (LHK15) (**B**) and counterstained with DAPI. All slides were photographed at the same magnification using the Leica Epi DM5000B microscope (scale bar = 100 μm). Western blotting of keratins present in HaCaT, N/Tert-1, KEB-11 and NEB-1 cell lysates using anti-K14 LLOO1 and EPR1614Y for K15 (**C**) and LHK15 antibodies (**D**). GAPDH was used as loading control. Relevant bands were cropped from different blots and grouped together. Original blots are shown in supplementary Figs S2 and S3.

To further compare the specificity of the two antibodies, we investigated the specificity of LHK15 and EPR1614Y in K15+ and K15- epithelial cells including HaCaT (low level of K15)^9^, N/Tert-1 (K15+)^31^, NEB-1 (K15-)^33^, KEB-11 (K15-)^33^ and MCF-7 (K15-)^32^, grown in 2-D cultures by immunostaining and western blotting. As shown in Fig. 3A, we observed strong reactivity of EPR1614Y with keratin cytoskeleton present in all 5 cell lines tested (HaCaT, N/Tert-1, NEB-1, KEB-11 and MCF-7). However, on western blot EPR1614Y recognised the K15 band only in HaCaT and N/Tert-1 but not in NEB-1, KEB-11 or MCF-7 (Figs 1Bi and 3C). As a control the anti-K14 LLOO1 reacted with HaCaT, N/Tert-1, NEB-1 but not with KEB-11 as it was derived from a patient suffering from a recessive form of epidermolysis bullosa simplex (EBS) with a premature termination codon in the K14 gene (Fig. 3C).

We also tested the reactivity of LHK15 with all 5 cell lines and observed weak reactivity with HaCaT and strong reactivity with N/Tert-1 but no reactivity with NEB-1, KEB-11 or MCF-7 cells (Fig. 3B). On western blot LHK15 showed reactivity only with HaCaT and N/Tert-1 but not with NEB-1, KEB-11 or MCF-7 (Figs 1Bii and 3D).

### LHK15 and EPR1614Y immunoreactivity with differentiating keratinocytes in organotypic cultures (OTCs)

**Figure 4:**
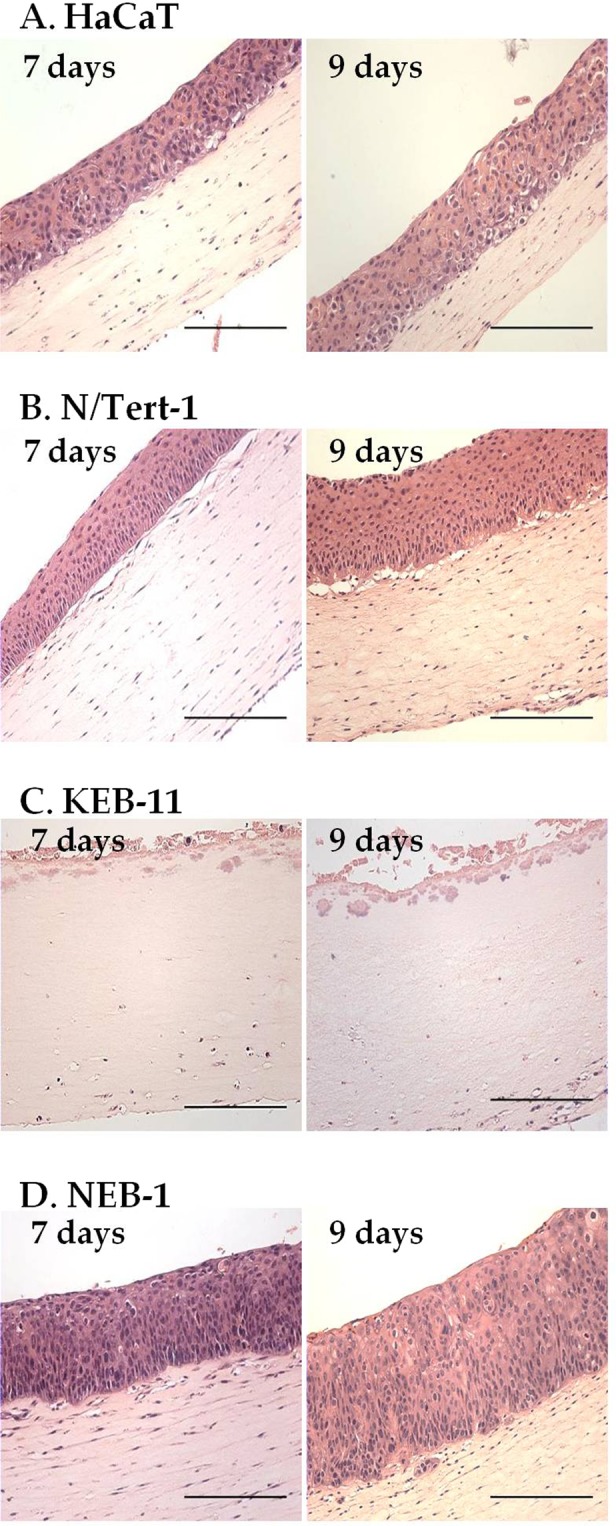
Organotypic cultures (OTCs) of keratinocyte cell lines. HaCaT (**A**), N/Tert-1 (**B**), KEB-11 (**C**) and NEB-1 (**D**) cells were co-cultured with primary dermal fibroblasts in OTCs for 7 and 9 days, followed by paraffin embedding and sectioning. Images of H&E-stained specimens were acquired using Leica Epi DM5000B or Nikon Eclipse 80i microscopes; scale bar = 200 μm.

To compare reactivity of the two antibodies in differentiating keratinocytes we prepared OTCs from HaCaT, N/Tert-1, KEB-11 and NEB-1. As shown in Fig. 4, only 3 cell lines HaCaT, N/Tert-1 and NEB-1 produced stratified epithelium, which as expected, was thicker after 9 days of culturing compared with that obtained after 7 days. KEB-11 cells did not produce a stratified epithelium under these conditions (Fig. 4).

**Figure 5:**
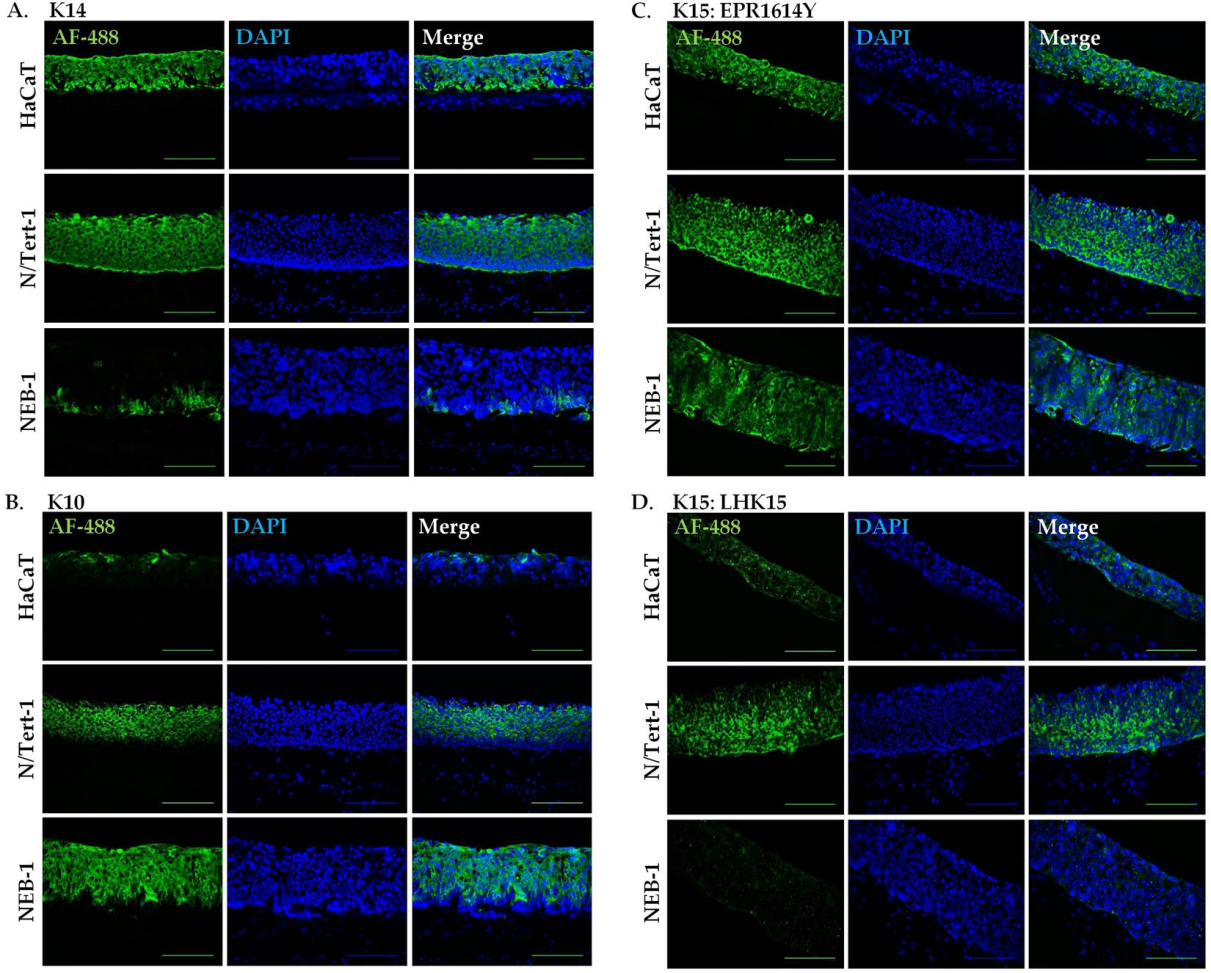
Immunoreactivity of OTCs with different antibodies. After 9 days of culturing HaCaT, N/Tert-1 and NEB-1 OTCs were cryofixed, sectioned and stained for K14 (**A**), K10 (**B**); K15 (EPR1614Y) (**C**); and K15 (LHK15) (**D**). The nuclei were counterstained with DAPI. Images were acquired using the Leica DM5000B epi-fluorescence microscope (scale bar = 200 μm).

To characterise the OTCs we immunostained the cryofixed sections of 3-D cultures for K14, a marker of basal keratinocytes^1^, and K10, a marker of suprabasal layers^32^. The expression of K14 was more or less homogenous in the stratified layers of OTCs from HaCaT and N/Tert-1. In NEB-1 cultures, K14 was expressed in patches of cells located near the junction of epithelium and the collagen base (Fig. 5A), whereas K10 expression was primarily located towards the suprabasal layers in OTCs from all 3 cell lines (Fig. 5B). In OTCs of HaCaT only few cells located at the top showed K10 expression whereas in N/Tert-1 and NEB-1 strong expression was seen in all suprabasal layers (Fig. 5B).

We then investigated the reactivity of EPR1614Y and LHK15 with cryofixed sections of OTCs derived from HaCaT, N/Tert-1 and NEB-1. EPR1614Y reacted strongly with all layers of OTCs derived from the 3 different cell lines (Fig. 5C). LHK15 on the other hand reacted weakly with HaCaT OTCs but it reacted strongly with N/Tert-1 OTCs (Fig. 5D). The most significant difference was the strong reactivity of EPR1614Y with NEB-1 derived OTCs, whereas LHK15 showed no reactivity with these OTCs (Fig. 5C,D).

### K15 add back experiments to restore antibody reactivity

**Figure 6:**
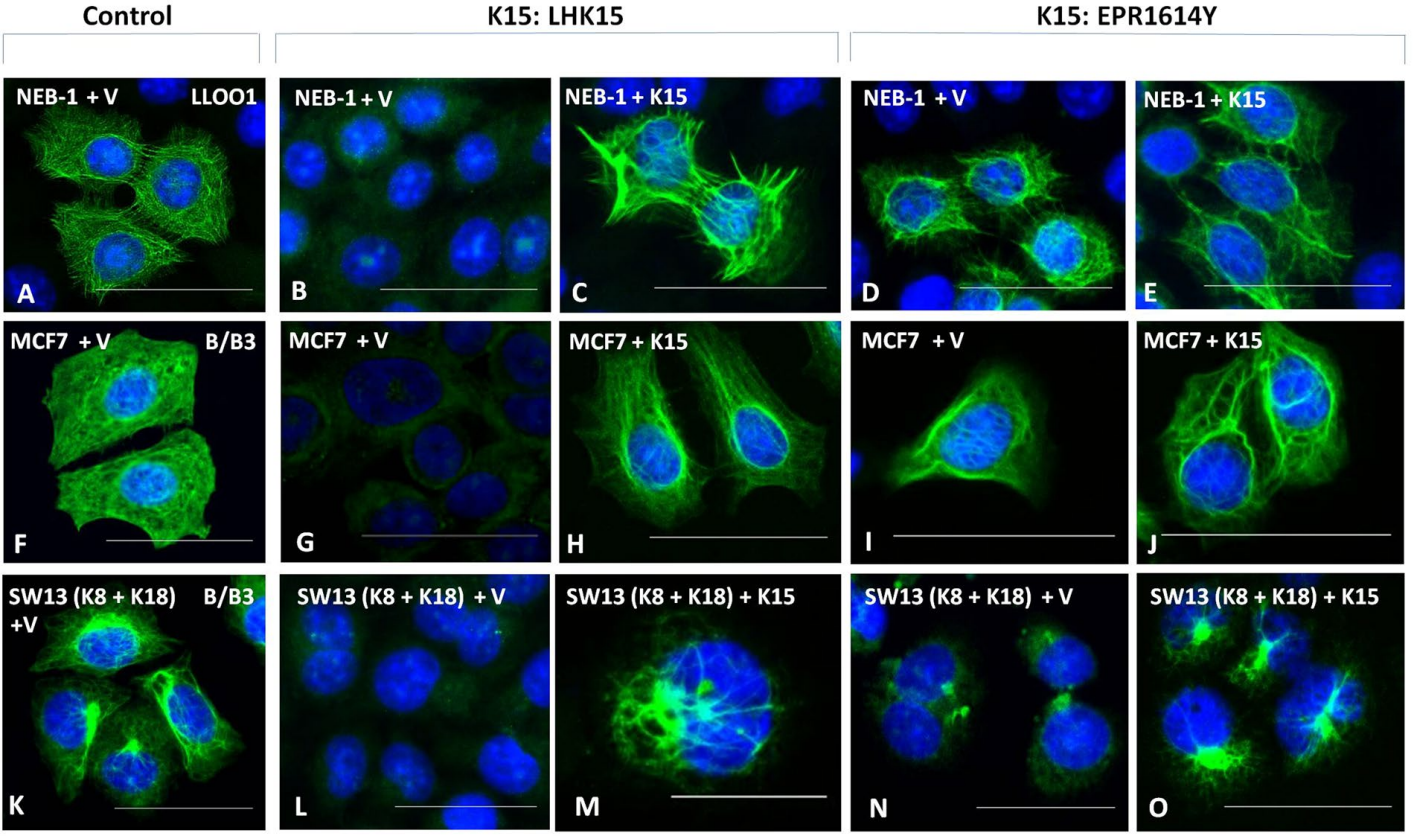
Immunostaining of epithelial cells with LHK15 and EPR1614Y in the presence and absence of K15 polypeptide. NEB-1 and MCF-7 cells were transduced with vector control and K15 recombinant retroviruses, after puromycin selection they were immunostained with LHK15 and EPR1614Y. NEB-1 vector control cells stained for K14 (LLOO1) (**A**), K15 (LHK15) (**B**), and K15 (EPR1614Y) (**D**), NEB-1 cells transduced with K15 virus were stained for K15 (LHK15) (**C**) and K15 (EPR1614Y) (**E**). MCF-7 vector control cells stained for K8 (A45-B/B3) (**F**), K15 (LHK15) (**G**) and K15 (EPR1614Y) (**I**), MCF-7 cells transduced with K15 virus were stained for K15 (LHK15) (**H**) and K15 (EPR1614Y) (**J**). SW13 cells which do not express any keratin were transduced with K8 and K18 retroviruses and selected with G418 and hygromycin. SW13 (K8 + K18) cells were transduced with vector control virus and after selection with puromycin stained for K8 (A45-B/B3) (**K**), K15 (LHK15) (**L**) and K15 (EPR1614Y) (**N**), SW13 (K8 + K18) cells transduced with K15 virus were stained for K15 (LHK15) (**M**) and K15 (EPR1614Y) (**O**). All images were acquired at the same gain and exposure using the Leica DM4000B epi-fluorescence microscope (scale bar = 50 μm).

To further investigate the specificity, we tested reactivity of these antibodies after overexpressing K15 in cells devoid of this polypeptide. As K15 is absent in NEB-1 cells (Fig. 3B–D), we ectopically expressed K15 in these cells using retroviral transduction and immunostained the cells with LHK15 and EPR1614Y. As shown in Fig. 6A the NEB-1 cells transduced with the vector control virus immunoreacted with anti-K14 LLOO1 but did not stain with LHK15 (Fig. 6B) whereas the K15 transduced cells stained strongly with this antibody (Fig. 6C). EPR1614Y antibody on the other hand reacted strongly with NEB-1 in the absence as well as in the presence of K15 (Fig. 6D,E). These observations suggest that EPR1614Y may be reacting non-specifically with keratin filaments in the absence of K15.

We then did a similar experiment using MCF-7 cells, which express only K8, K18 and K19^1,32^. Vector control MCF-7 cells reacted with K8 antibody A45-B/B3 (Fig. 6F), they did not react with LHK15 (Fig. 6G) but the reactivity was induced when K15 was added into MCF-7 cells (Fig. 6H). On the other hand, EPR1614Y reacted strongly with MCF-7 vector control cells (Fig. 6I) which did not change when K15 was added into these cells (Fig. 6J).

To substantiate the above observations, we transduced SW13, a cell line which does not express keratin filaments, with recombinant retroviruses expressing K8 and K18 and applied double selection with G418 and hygromycin. K8 and K18 formed IFs in SW13 cells as shown by A45-B/B3 immunostaining (Fig. 6K), these IFs did not react with LHK15 (Fig. 6L). SW13 cells expressing K8 + K18 [SW13(K8 + K18)] showed weak but detectable reactivity with EPR1614Y (Fig. 6N). The SW13(K8 + K18) cells were further transduced with K15 containing retroviruses and they were subjected to triple selection with G418, hygromycin and puromycin. As expected, the immunoreactivity of LHK15 with SW13(K8 + K18) was induced when K15 was added into these cells (Fig. 6M) and the reactivity of EPR1614Y with these cells became stronger and more filamentous in the presence of K15 (Fig. 6O). Taken together these results suggest that the reactivity of LHK15 is specific to K15 whereas EPR1614Y will react non-specifically with other keratins in the absence of K15 polypeptide.

## Discussion

Immunochemistry, flow cytometry, enzyme-linked immunosorbent assays (ELISAs) and western blotting provide some of the most valuable, powerful, research as well as diagnostic tools in the field of biomedical sciences. However, it is well-known that the choice of the most specific and reliable antibody is of critical importance, as it determines the validity and credibility of the scientific data. Use of a non-specific antibody could lead to false results and conclusions^34^. As an example, a 2009 editorial by Michel and co-workers demonstrated that lack of selectivity appears to be the rule rather than the exception for antibodies against G-protein-coupled receptors (49 antibodies against 19 subtypes of the receptors have been tested in those studies and none of them was found to be selective)^35^. Other authors had to withdraw their publications because the antibodies they used against novel markers were later found to react in tissues from knock-out mice that lacked the markers^36^. Hence, determining the specificity of antibodies is extremely important especially when the antigen, K15, is widely used as a biomarker for adult stem cells^37–42^.

Before the use of synthetic peptides for monospecific antibody production became common, most of the antibodies raised were against keratin complexes derived from cytoskeletal extracts or using purified peptides from SDS gels. Some of these antibodies recognised keratin filaments only under certain physiological conditions. For example, AE1 and COU-1 react only with tumour cells^43,44^ and K_G_8.13 reacts with cells undergoing mitosis^45^. A number of monoclonal antibodies such as K_s_18.18, LE61 and LE65 do not react with individual polypeptide but instead their epitopes are reconstituted only when complementary keratins associate into heterotypic complexes^46–48^. Other antibodies, such as A45-B/B3, are able to recognise conformational changes associated with heterotypic association of keratin pairs^49^. On the other hand, most antibodies raised using synthetic peptides reacted strongly with specific keratins on western blots as well as immunochemistry which increased their usefulness in keratin expression studies. Here we have compared the reactivity of two of the most widely used monoclonal antibodies, LHK15 and EPR1614Y, for the stem cell biomarker K15 by western blotting and immunochemistry in 2-D and 3-D cultures.

The choice of K15 antibody to address a particular set of research questions should be decided primarily by the techniques to be employed. On western blotting both antibodies, LHK15 and EPR1614Y, reacted strongly with K15 polypeptides in N/Tert-1 but no reactivity was observed with any protein band in MCF-7 cells suggesting they both specifically recognise a linear or denatured epitope. Therefore, if western blotting assay for K15 expression is to be used then either of the antibodies could be employed. However, if the expression of K15 polypeptide is to be investigated in cells and tissues by immunochemistry then significant differences in the ability of LHK15 and EPR1614Y to recognise K15 were observed. In the epidermis both LHK15 and EPR1614Y reacted with the basal keratinocytes, however LHK15 gave discontinuous reactivity which was consistent with the data reported previously^8,9^, EPR1614Y on the other hand gave a homogenous reactivity with both foreskin as well as abdominal skin. This is in variance with a recent report where EPR1614Y was shown to recognise patches of basal keratinocytes in human breast skin^50^. It is therefore conceivable that patchy vs continuous staining of K15 depends on the body site where skin was taken for immunohistochemistry. Although the molecular basis for the discontinuous staining of adult skin section by LHK15 is not clear, it could be due to the heterogeneity in the basal keratinocytes reported previously^51–53^. This antibody has been shown to give a continuous staining in foetal skin as well as internal epithelia such as oral and oesophageal epithelia^8,9^.

On immunocytochemistry the IFs in the human epidermal keratinocyte cell lines, NEB-1, KEB-11 and N/Tert-1, reacted very differently with LHK15 and EPR1614Y. On western blotting, both LHK15 and EPR1614Y did not react with any protein band in lysates of NEB-1 and KEB-11 suggesting they did not express K15 (Fig. 3C,D). The absence of K15 in NEB-1 and KEB-11 could be due to HPV16 induced immortalisation, perhaps involving post-transcriptional mechanisms^33^. Absence of K15 in KEB-11, which already does not express K14, would leave no type I basal keratin which explains why this cell line does not form stratified epithelia in OTCs (Fig. 4C). Surprisingly, EPR1614Y gave positive reactivity with all epithelial cell lines used in this study whereas LHK15 reacted only with HaCaT and N/Tert-1 (Figs 3, 5 and 6). Absence of immunofluorescence reactivity of LHK15 with KEB-11, NEB-1 and MCF-7 suggests that presence of K15 is an absolute requirement for this antibody to show reactivity. EPR1614Y on the other hand reacted with keratin cytoskeleton even in the absence of K15, which implies that the three-dimensional reconstitution of EPR1614Y epitope may not require presence of K15.

The epitope of LHK15 is derived from the last 17 residues of K15 polypeptide which is the end of the tail domain (Fig. 1A), a region which can vary strongly in size and sequence amongst keratins^54,55^. Therefore, an antibody raised against this region is most likely to be specific towards its target. The epitope for EPR1614Y is poorly defined. This antibody was generated by Abcam UK using their own patented technology and their website claimed the epitope was between residues 400–500, however, as there are only 456 residues in K15 polypeptide, the epitope must be between 400–456 residues. Assuming the epitope begins from residue 400 in K15 polypeptide, the peptide used to raise this antibody would contain the last 13 residues of the rod domain. This region is part of the Helix Termination Peptide (HTP), which is highly conserved and it is a high affinity association site for stabilisation of heterotypic complexes between type I and type II keratins^56^. Mutations in this region are known to destabilise the cytoskeleton and are associated with a large number of blistering syndromes^57–59^. The high degree of sequence conservation in this region amongst different keratins^56^ suggests that heterotypic keratin complexes could assemble into a very similar three-dimensional epitope even in the absence of K15. This hypothesis is supported by the data presented in Fig. 6 which shows that EPR1614Y reactivity in the absence of K15 seems to depend on the number of polypeptides present in the cytoskeleton, with low reactivity when K8 + K18 were present and increasing strongly in MCF-7, with additional keratin K19, and in NEB-1 which would express other keratins such as K5, K14, K1 and K10. This explains why EPR1614Y reacts with keratin cytoskeleton devoid of K15 in immunocytochemistry but will not react on western blot if K15 was absent. There are other reports in the literature where cytoskeleton formed from different IF polypeptides are recognised by the same antibody. The well-documented example is that of anti-IFA antibody which recognises intermediate filaments formed from almost all IF polypeptides including keratins, vimentin, desmin, neurofilaments and glial fibrillary acidic protein^60^. Interestingly the epitope of this antibody is also located in the highly conserved HTP region^49,61,62^.

The antibody LHK15 reacts with K15 from human, rat, cow and mouse (www.abcam.com/cytokeratin-15-antibody-lhk15-ab80522.html) whereas EPR1614Y reacts only with mouse and human (www.abcam.com/cytokeratin-15-antibody-epr1614y-ab52816.html) but both reagents react strongly with keratin filaments fixed with either acetone/methanol or with formalin. As the epitope for EPR1614Y is not defined, it is conceivable that the epitope does not involve HTP and it is derived entirely from the tail domain. In that case it is possible that during heterotypic dimerisation the tail domains of type I and type II keratins associate such that they form a 3-D structure mimicking the EPR1614Y epitope.

The EPR1614Y antibody has been widely used to study K15 expression as a biomarker of epidermal stem cells in scar formation^63^, differentiation of human hair follicle cells into neurons^64^, psoriatic skin lesions^50^, ameloblastoma^65^, mammary epithelium^66^, developing ureters^67^ and human cornea^37^. In view of our data that EPR1614Y would react with cell cytoskeleton even when K15 is absent, some of the published work in which this antibody has been used may require reinterpretation. Furthermore, as our understanding of the clinical relevance of K15 positive cells in cancer, tissue homeostasis and other pathology develops, it is fundamental that only highly specific antibodies are employed to correctly identify these important cells *in vivo*.

In conclusion, we show that both antibodies, LHK15 and EPR1614Y, recognise K15 on western blotting. However, in immunocytochemistry LHK15 showed reactivity only when K15 was present whereas EPR1614Y showed strong reactivity even when there was no K15 polypeptide. The study highlights the extreme care one should exercise in choosing and validating the specificity of an antibody for their studies.

## Methods and Materials

### Materials

The cell lines and antibodies used along with their sources in parenthesis were as follows: HaCaT, KEB-11 and NEB-1 (Cancer Research United Kingdom Laboratories, CRUK), MCF-7 (in house), N/Tert-1 (James Rheinwald, Harvard Medical School, Boston, USA), Phoenix E (Gary Nolan, Stanford University), PT67 (Dusty Miller, Fred Hutchinson Cancer Research Centre, University of Washington, USA), A45-B/B3, a gift from Uwe Karsten (Max Delbruck Centre for Molecular Medicine, Berlin-Buch, Germany) and anti-K14 LLOO1 (CRUK laboratories). DMEM (Lonza), FCS (First Link UK Ltd), penicillin and streptomycin (Gibco^®^, Life technologies Ltd) and components of the FAD medium were all obtained from Sigma-Aldrich apart from Ham’s F12, which was obtained from Lonza. The commercially available primary antibodies used in this study were as follows: anti-GAPDH (ab9485), mouse anti-K15 clone LHK15 (ab80522), rabbit anti-K15 clone EPR1614Y (ab52816) and anti-K10 clone RKSE60 (ab902) from Abcam, UK. The secondary antibodies used were as follows: peroxidase goat anti-mouse (AP124P) and goat anti-rabbit (AP132P) IgGs (Millipore, UK) and Alexa Fluor 488-labeled goat anti-mouse IgG (H + L) (A11029) and F(ab′)2-goat anti-rabbit IgG (H + L) (A11070) from Life Technologies, UK. Retroviral vector pLPC-N MYC was a gift from Professor Titia de Lange (Addgene plasmid # 12540). Informed consents were obtained from all patients for collection of skin samples which was reviewed and approved by the NRES Committee London- City and East (REC ref. 09/H0704/69) and all methods were performed in accordance with the relevant guidelines and regulations.

### Molecular cloning

All molecular cloning experiments were performed using standard cloning techniques^68^. The retroviral expression vector pLPC_NMyc was re-engineered to remove N-Myc tag and replaced by K15 cDNA allowing its expression to be driven by CMV promoter. Total mRNA was isolated from K15 + N/Tert-1 cells using the Dynabeads^®^ mRNA DIRECT™ Purification Kit (Ambion^®^, Paisley, UK) and transcribed into cDNA by a mixture of poly A and random hexamer using the qPCRBIO cDNA Synthesis Kit (PCRBIOSYSTEMS, PB30.11–10) according to the manufacturer’s instructions. K15 cDNA was amplified using Q5 DNA polymerase (New England Biolabs, UK) and ligated into *Eco*RI and *Bam*HI sites of pLPCpuro. Keratin K18 cDNA was ligated in *Eco*RI and *Hin*dIII sites of pLPCneo and K8 cDNA was ligated in *Eco*RI and *Bam*HI sites of pLPChygro vectors.

### Cell culture

Phoenix E (ecotropic), PT67 (amphotropic) retroviral packaging cell lines and HaCaT, a spontaneously immortalised normal epidermal keratinocytes^69^, were cultured in DMEM with 10% (v/v) FCS with 100 units/ml penicillin and 100 μg/ml streptomycin (1 x Pen/Strep). N/Tert-1, normal epidermal keratinocytes immortalised by overexpression of h-Tert and downregulation of p16^70^, KEB-11, K14 deficient keratinocytes derived from an EBS patient^71,72^ and NEB-1, derived from an unaffected relative of the EBS patient used as a control for KEB-11, both immortalised by HPV16^73^ were cultured in FAD medium consisting DMEM and Ham’s F12 in 3:1 ratio containing 10% FCS, 1 X Pen/Strep, adenine (24 μg/ml), cholera toxin (8.4 ng/ml), epidermal growth factor (10 ng/ml), hydrocortisone (0.4 μg/ml), insulin (5 μg/ml), liothyronine (2 × 10^−11^ M) and transferrin (5 μg/ml)^74^. All cells were cultured at 37 °C in a humidified atmosphere of 5–10% CO_2_.

### Retroviral packaging, viral transduction using spinfection

Phoenix E cells were transfected with the constructs pLPCneo, pLPChygro and pLPCpuro (control vectors) and pLPCneo_K18, pLPChygro_K8 and pLPCpuro_K15 using *TransIT*^®^-*LT1* transfection reagent (Cambridge Biosciences, UK). After 48 h culture supernatants containing ecotropic viruses were used to transduce PT67 cells in presence of 5 μg/ml polybrene (1,5-dimethyl-1,5-diazaundecamethylene polymethobromide) using the spinning method as described previously^31,75^. Briefly, the viral supernatant was treated with 5 μg/ml polybrene and the growth medium was replaced with the prepared viral supernatant. Plates were centrifuged at 1000 rpm at 32 °C for 1 h and then incubated for 24 h at 32 °C in a humidified atmosphere of 10% CO_2_. Cells were cultured in fresh complete growth medium at 37 °C for 48 h to ensure viral integration and protein expression followed by appropriate drug selection (1 mg/ml G418; 400 µg/ml hygromycin and 1–1.5 µg/ml puromycin) for several days. Amphotropic retroviral supernatants were collected from PT67 cells three times every 24 h and snap frozen and stored at −80 °C. SW13 cells were transduced with K18 followed by K8 amphotropic retrovirus and selected with a combination of G418 and hygromycin. MCF-7, SW13 (K8 + K18) and NEB-1 cell lines were transduced with K15 (or control) amphotropic retroviral supernatant using the spinfection method as described above and selected with puromycin.

### 3-dimensional tissue culture model of keratinocyte differentiation

HaCaT, N/Tert-1, KEB-11 and NEB-1 keratinocytes were seeded on a human primary dermal fibroblast containing disc of rat-tail collagen type I (Corning, UK) in a Millipore^®^ Millicell^®^ (Sigma-Aldrich, UK). The cells were cultured for 24 h at 37 °C in a humidified atmosphere of 10% CO_2_ and maintained in FAD medium. After 24 h the collagen disc was raised to the air-liquid interphase to induce keratinocyte differentiation. The cells were cultured for 7 or 9 more days after which they were either fixed with 4% (w/v) paraformaldehyde, embedded in paraffin or cryofixed and sectioned for immunostaining.

### SDS electrophoresis and western blotting

Cultured cells were lysed in lysis buffer (4% (w/v) SDS, 20% (v/v) glycerol in 0.125 M Tris HCl, pH 6.8), heated to 95 °C for 5 min and the protein concentration was measured with *DC™ Protein Assay* (Bio-Rad, Hemel Hempstead, UK). Just before electrophoresis, 10% (v/v) 2-mercaptoethanol and 0.004% (w/v) bromophenol blue were added and the lysate was separated by SDS-PAGE (NuPAGE^®^ Novex^®^ 10% Bis-Tris Protein Gels) with NuPAGE^®^ MOPS SDS Running Buffer (Novex^®^, Paisley, UK). The proteins in the gel were transferred onto 0.45 μm nitrocellulose membranes (Sigma-Aldrich, Gillingham, UK) in transfer buffer (20 mM Tris base, 190 mM glycine, 20% (v/v) methanol). The membranes were blocked with 5% (w/v) non-fat dry milk for 30 min and washed with TBS-T buffer (20 mM Tris base, 150 mM NaCl, 1% (v/v) Tween^®^20). Membranes were probed with primary antibodies overnight at 4 °C and incubated with the secondary antibody for 1 h at room temperature (RT). The target proteins were detected using Amersham ECL Prime Western Blotting Detection Reagent (GE Healthcare, Little Chalfont, UK). Peroxidase activity was detected with Amersham Hyperfilm™ ECL autoradiography film (GE Healthcare).

### Immuno-cytochemistry and –histochemistry

Cells were cultured on collagen-coated coverslips for 24 h, fixed in a mixture of ice-cold acetone and methanol (1:1) for 10 min and dried in air. After blocking the non-specific antibody reactivity with 10% (v/v) normal goat serum, the coverslips were incubated with primary antibody for 2 h at RT. After washing with phosphate-buffered saline (PBS) the coverslips were incubated with Alexa Fluor^®^ 488-labelled anti-mouse or anti-rabbit secondary antibodies in the dark for 1 h at RT. The coverslips were washed again and mounted on glass slides using VECTASHIELD HardSet Antifade Mounting Medium with DAPI (Vector Laboratories, UK) and visualised with a Leica Epi DM5000B or DM4000B microscope equipped with a DFC350 FX digital camera and 20x/0.5 NA and 40x/0.75 NA objective lenses.

The 3-D reconstructed plugs or human tissues were cryo-sectioned and attached to glass slides, fixed in ice-cold methanol for 15 min and washed with PBS. The sections were treated with primary antibodies at 4 °C overnight, washed, stained with secondary antibody and mounted in DAPI containing mounting medium as described above for cultured cells.

## Supplementary information

Supplementary figures S1, S2 and S3

## Data Availability

Any supporting data not included in this manuscript or reagents used in this study, which are not commercially available, will be provided to readers following a written request to the corresponding author.
