# Supplementary material for: The monoclonal antibody EPR1614Y against the stem cell biomarker keratin K15 lacks specificity and reacts with other keratins
Source: Sci Rep. 2019 Feb 13;9:1943. doi: 10.1038/s41598-018-38163-5 (PMC6374370; doi:10.1038/s41598-018-38163-5)
Supplement: Supplementary file 1 — Supplementary figures S1, S2 and S3 [file 41598_2018_38163_MOESM1_ESM.pdf]

The monoclonal antibody EPR1614Y against the stem cell biomarker keratin K15 lacks specificity and reacts with other keratins

Hebah Aldehlawi, Katarzyna A. Niemiec, Deepa R Avisetti, Anand Lalli, Muy-Teck Teh, and Ahmad Waseem

Supplementary Information

(i)

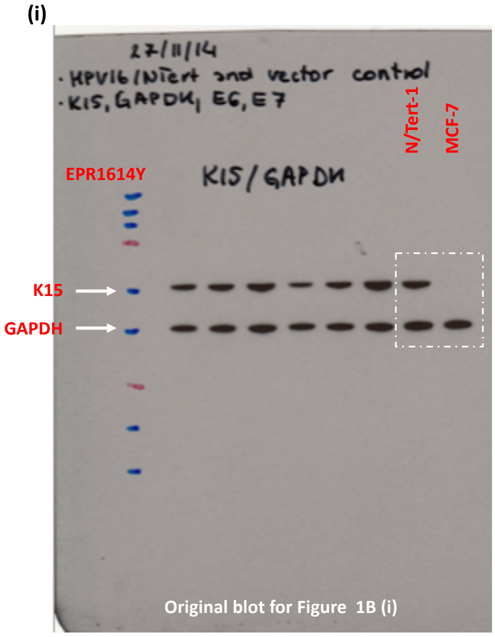

(ii)

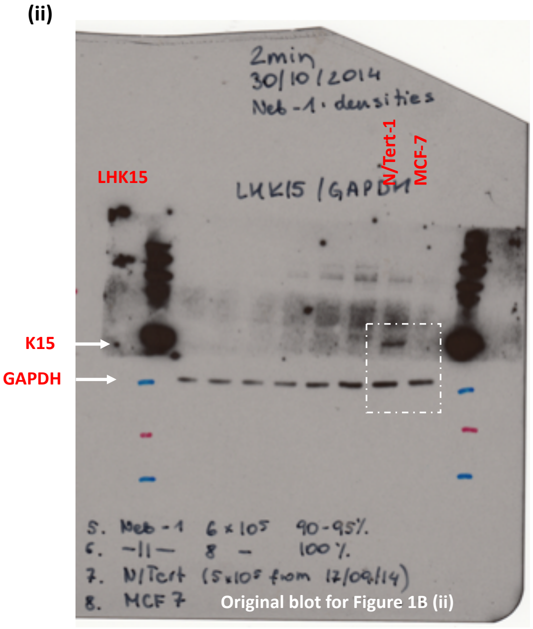

Supplementary figure 1: Raw western blot data corresponding to the cropped blots in the figure 1B. Western blotting of keratins extracted from N/Tert-1 and MCF-7 using EPR1614Y (i) and LHK15 (ii). GAPDH was used as loading control. The cropped area is shown in inset.

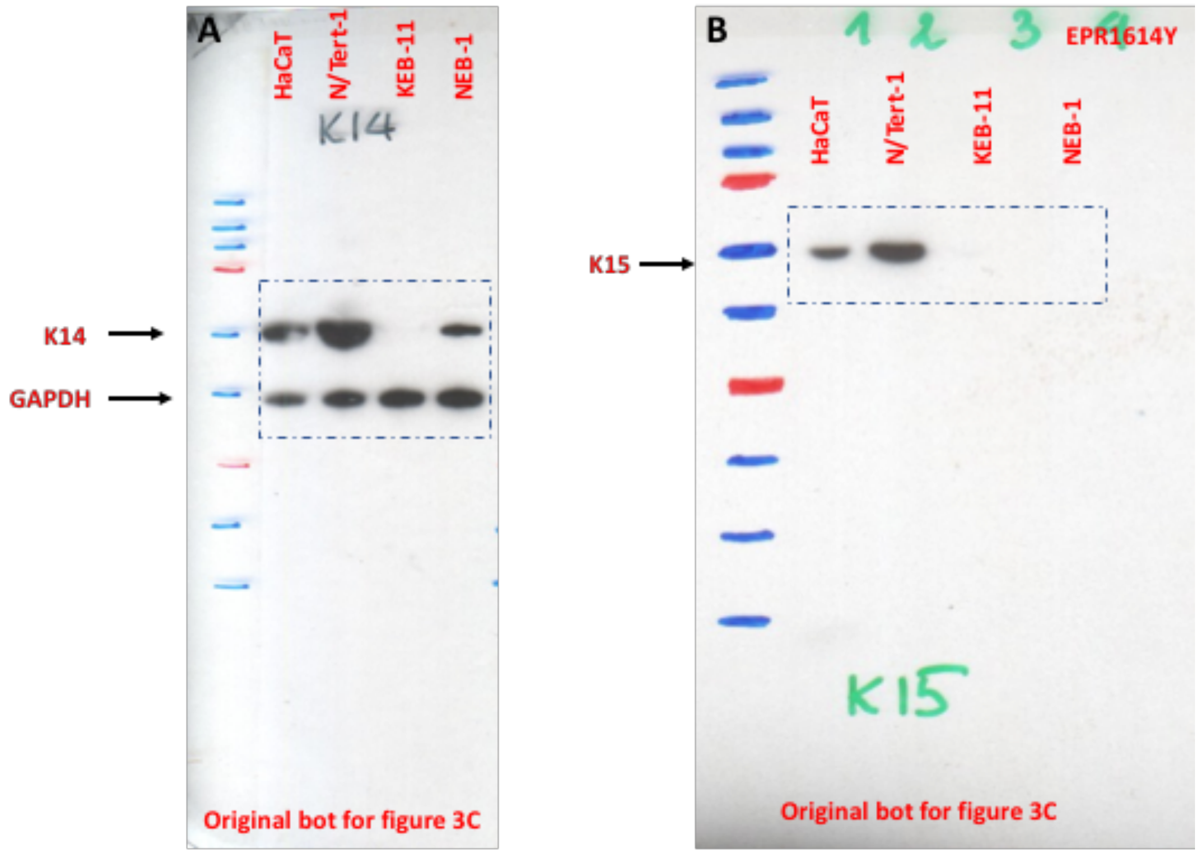

Supplementary figure 2: Raw western blot data corresponding to cropped blots in the figure 3C. Western blotting of keratins extracted from HaCaT, N/Tert-1, KEB-11 and NEB-1 cells using K14 antibody LLOO1 (A), K15 antibody EPR1614Y (B). GAPDH was used as loading control. The cropped area is shown in inset

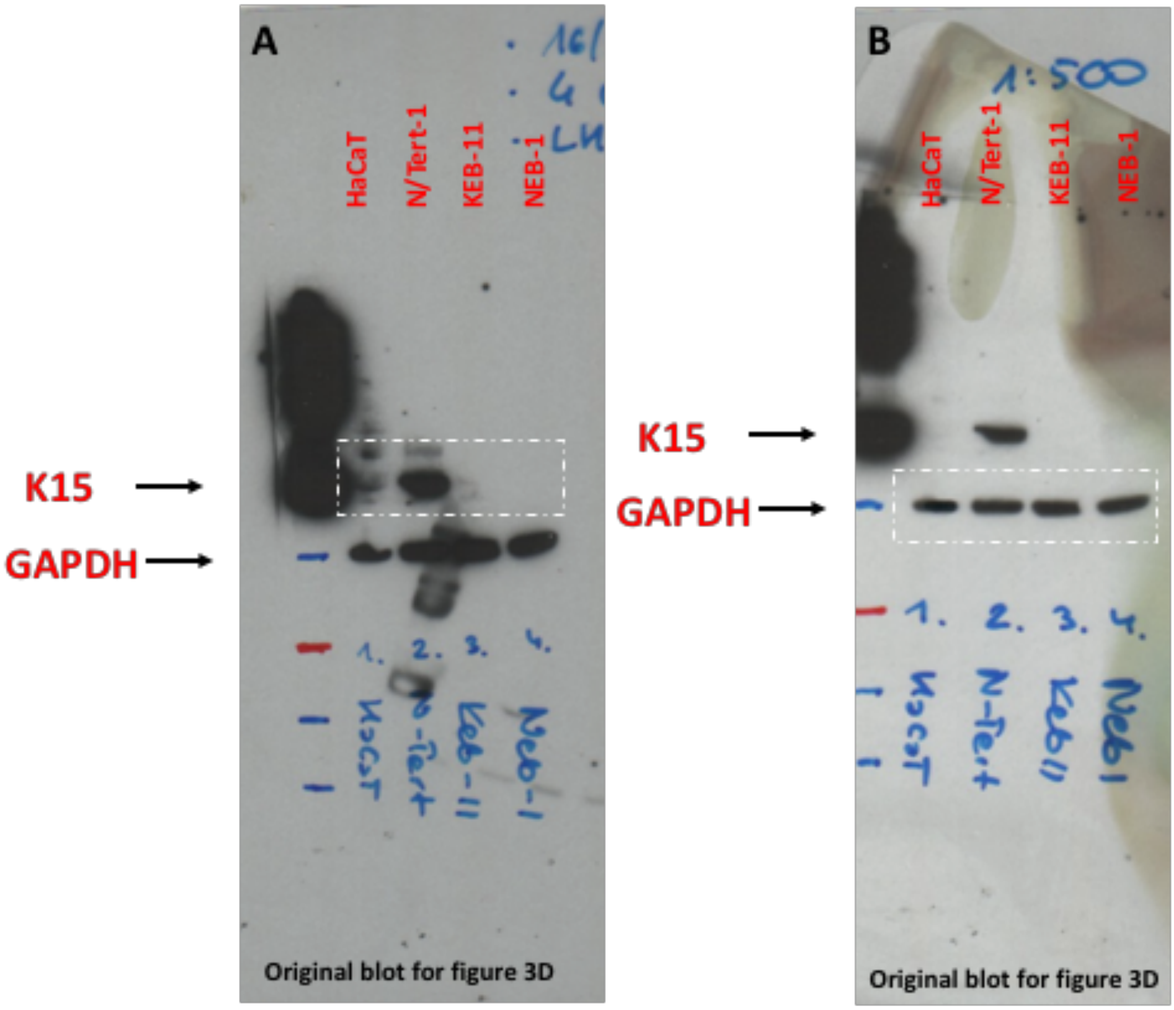

Supplementary figure 3: Raw western blot data corresponding to the cropped blots in the figure 3D. Western blotting of keratins extracted from HaCaT, N/Tert-1, KEB-11 and NEB-1 cells using LHK15 antibody. GAPDH was used as loading control. (A) Exposure for LHK15 and (B) exposure for GAPDH.
